# Metabolic syndrome, diabetes and inadequate lifestyle in first-degree relatives of acute myocardial infarction survivors younger than 45 yrs. old

**DOI:** 10.1186/1758-5996-7-S1-A161

**Published:** 2015-11-11

**Authors:** Maria Helane da Costa Gurgel Castelo, Renan Magalhães Montenegro, Clarisse Mourão Melo Ponte, Tamara Cristina S Sousa, Paulo Goberlanio B Silva, Lucia de Sousa Belém, Alexandre C Pereira, Raul Dias Santos Filho

**Affiliations:** 1 Faculdade de Medicina da universidade Federal do Ceará, Fortaleza, Brazil

## Background

Acute Myocardial infarction (AMI) before the age of 45 is unusual and is associated with a familial component.This study evaluated cardiovascular risk factors in a cross-sectional study of first-degree relatives of Brazilian patients with premature MI.

## Materials and methods

A total of 166 first-degree relatives (FDR) of 103 patients with MI age <45 yrs. were matched for sex and age with a group of 111 individuals with no family history of cardiovascular disease (control group). Familial hypercholesterolemia was excluded. Patients were evaluated for the presence of metabolic syndrome, its components, and lifestyle (smoking, alcohol consumption, and sedentarism). Laboratory analysis included fasting blood glucose, plasma lipids and thyrotropin (TSH).

## Results

The prevalences of smoking (29.5 vs. 6.3%, p<0.001), prediabetes (40.4 vs. 27, p<0.001), diabetes (19.9 vs. 1.8%, p<0.001), metabolic syndrome (64.7 vs. 36%, p<0.001), and dyslipidaemia (84.2 x 31.2%, p: 0.001) were higher in FDR individuals. Triglycerides (179±71 vs. 140±74mg/dL, p: 0.002), LDL-cholesterol (122±36vs.113±35mg/dL, p: 0.031), non-HDL cholesterol (157±53 vs.141±41mg/dL, p: 0.004), and TSH levels (2.4±1.6 vs. 1.9±1.0, p: 0.002) were also higher, and HDL-cholesterol (39±10 vs. 48±14mg/dL, p<0.001) lower in FDR. No significant differences were observed between groups for body mass index, abdominal obesity, hypertension, total cholesterol, and fasting blood glucose levels.

## Conclusions

FDR of patients with AMI < age of 45 yrs. old without familial hypercolesterolemia present elevated prevalence of the metabolic syndrome and its components, as well as an inadequate lifestyle.

